# Exploring cancer metastasis prevention strategy: interrupting adhesion of cancer cells to vascular endothelia of potential metastatic tissues by antibody-coated nanomaterial

**DOI:** 10.1186/s12951-015-0072-x

**Published:** 2015-02-03

**Authors:** Jingjing Xie, Haiyan Dong, Hongning Chen, Rongli Zhao, Patrick J Sinko, Weiyu Shen, Jichuang Wang, Yusheng Lu, Xiang Yang, Fangwei Xie, Lee Jia

**Affiliations:** Cancer Metastasis Alert and Prevention Center, and Biopharmaceutical Photocatalysis of State Key Laboratory of Photocatalysis on Energy and Environment, College of Chemistry, Fuzhou University, 523 Industry Road, Science Building, 3FL, Fuzhou, Fujian 350002 China; Rutgers, The State University of New Jersey, 160 Frelinghuysen Road, Piscataway, NJ 08854 USA; Department of Medicine Oncology, East Hospital of Xiamen University, Fuzhou, 350004 China

**Keywords:** Cancer metastasis prevention, Circulating tumor cells, Antibody conjugation, Multivalent binding, Cell adhesion

## Abstract

**Background:**

Cancer metastasis caused by circulating tumor cells (CTCs) accounts for 90% cancer-related death worldwide. Blocking the circulation of CTCs in bloodstream and their hetero-adhesion to vascular endothelia of the distant metastatic organs may prevent cancer metastasis. Nanomaterial-based intervention with adhesion between CTCs and endothelia has not been reported. Driven by the novel idea that multivalent conjugation of EpCAM and Slex antibodies to dendrimer surface may enhance the capacity and specificity of the nanomaterial conjugates for capturing and down-regulating colorectal CTCs, we conjugated the dendrimer nanomaterial with the EpCAM and Slex antibodies, and examined the capacity of the dual antibody-coated nanomaterial for their roles in interrupting CTCs-related cancer metastasis.

**Results:**

The antibody-coated nanomaterial was synthesized and characterized. The conjugates specifically bound and captured colon cancer cells SW620. The conjugate inhibited the cells’ viability and their adhesion to fibronectin (Fn)-coated substrate or human umbilical vein endothelial cells (HUVECs) in a concentration-dependent manner. In comparison with SW480 and LoVo cell lines, the activity and adhesion of SW620 to Fn-coated substrate and HUVECs were more specifically inhibited by the dual antibody conjugate because of the higher levels of EpCAM and Slex on SW620 cell surface. The hetero-adhesion between SW620 and Fn-coated substrate, or HUVECs was inhibited by about 60-70%. The dual conjugate showed the inhibition capacity more significant than its corresponding single antibody conjugates.

**Conclusions:**

The present study provides the new evidence that coating nanomaterials with more than one antibody against CTCs may effectively interfere with the interaction between SW620 and HUVECs.

**Electronic supplementary material:**

The online version of this article (doi:10.1186/s12951-015-0072-x) contains supplementary material, which is available to authorized users.

## Background

Cancer is the second killer that leads people to death worldwide [1,2]. It was found that cancer metastasis was the principal cause of death among cancer patients [3-5]. The presence of circulating tumor cells (CTCs) [6-8], which are detached from primary tumor and enter the bloodstream [9], may contribute to initiate cancer metastasis. The progress of cancer metastasis usually depends on a series of consequential events, including the activation of dormant CTCs, the hetero-adhesion of CTCs to vascular endothelial bed of secondary organs, the continued survival and proliferation of CTCs after extravasation, and the formation of initial micrometastatic foci [10]. It seems that the effective prevention of cancer metastasis may be achieved by interrupting the circulation or activation of CTCs in blood and/or inhibiting the adhesion between CTCs and vascular endothelial cells.

CTCs as the hallmark of cancer metastasis have been paid more attention. To effectively interfere with the CTCs-related cancer metastasis, the residual CTCs should be preferentially captured and restrained with the enhanced specificity [11]. However, owing to the low number of CTCs in blood [12,13], capturing CTCs is a great technological challenge. Current chemotherapeutics and nanomaterials-based drug delivery system are designed to kill the malignant cancer cells, not CTCs *per se*. The serious adverse effects resulted in the damaged normal tissues and the decreased immunity [14,15]. Once CTCs in bloodstream were activated, cancer metastasis will be irrevocably initiated [16,17]. Some techniques were developed to capture CTCs, such as employing the epithelial cell adhesion molecule (EpCAM) antibody-coated three-dimensional nanostructured substrates [18,19], dendrimers [20], graphene oxide nanosheets [21] or immunomagnetic nanospheres [20,22,23]. However, these studies were only confined to functionalize nanomaterials with one targeting antibody against a single CTCs surface biomarker. Besides, the abundance of one biomarker varies dynamically with the cell cycle [24,25]. The level and activity of EpCAM expressed by CTCs not by hematologic cells was decreased with the epithelial-to-mesenchymal transition [26-28]. Therefore, the weak binding affinity of nanomaterials assembling one targeting antibody can't assure the extremely exact capture of CTCs. The unbound CTCs still made it possible to drive cancer metastasis.

Adhesion of CTCs to vascular endothelium was another crucial point of CTCs-derived cancer metastasis. Our previous studies demonstrated that chemopreventives such as S-nitrosocaptopril (CAP-NO) [10] and Metapristone (the metabolite of mifepristone) [29] with the low cytotoxicity had the intervention effects on the adhesion and invasion of colorectal CTCs to human umbilical vein endothelial cells (HUVECs). These related research laid the technical foundation for our study. Considering the fact that EpCAM [30,31] and saliva acidifying louis oligosaccharides (Sialyl Lewis X, Slex) [32,33] are over-expressed on the surface of colorectal CTCs in circulation [32]. EpCAM antibody (antiEpCAM) can directly interfere the adhesion process of CTCs [34] while Slex antibody (antiSlex) can indirectly block the adhesion between CTCs and endothelial cells through Slex/E-selection interaction [35,36]. Dendrimers-mediated multivalent binding effects were also exploited in previous studies [20]. Thus, we hypothesize that multivalent conjugation of both antiEpCAM and antiSlex to nanoscale polyamidoamine (PAMAM) dendrimers may significantly improve the anti-proliferation and anti-adhesion effects by enhancing the capture specificity, increasing the binding affinity, and avoiding the non-specific binding to similar cell subpopulations.

To test the hypothesis, we, herein, showed a novel strategy to realize the highly-specific binding, the restraint of colorectal CTCs, and the inhibition of adhesion of CTCs to vascular endothelial cells in vitro by using the bioconjugates that combine PAMAM dendrimers with dual targeting antibodies (antiEpCAM and antiSlex). Though attachment of both E-selectin and antiEpCAM to the functionalized glass substrates were previously reported [34], we, for the first time, showed conjugation of both antiEpCAM and antiSlex to dendrimers as one entity and its physicochemical characterization in this study. The dual roles of the bioconjugates in cancer metastasis prevention, including restraining the captured CTCs and inhibiting their adhesion, were also demonstrated here.

## Results

### Synthesis and physiochemical characterization of G6-5A-5S and PE-5A-G6-5S-FITC conjugates

**Figure 1:**
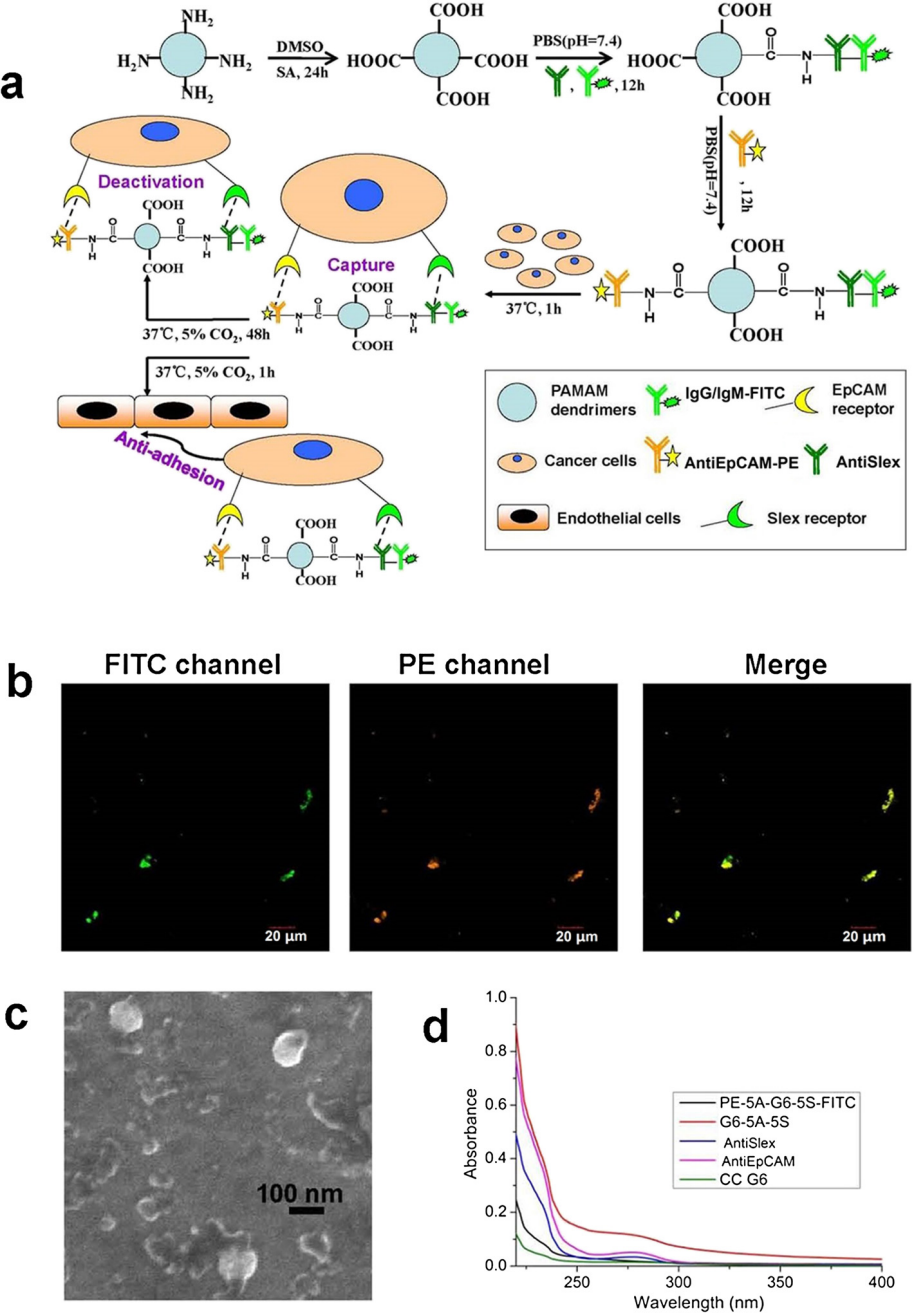
**Synthesis and physiochemical characterization of dendrimers assembling with antiEpCAM and antiSlex with or without fluorescence labeling. a**, Schematic illustration of the construction of dual antibody-conjugated dendrimers for exploring their biological functions in cancer metastasis through binding the target cancer cells. **b**, Images of PE-5A-G6-5S-FITC conjugate in aqueous solution under a laser confocal microscope. **c**, A FSEM image of the dry conjugate G6-5A-5S. **d**, The ultraviolet absorption spectra of CC G6 dendrimers, antibody and dual antibody conjugates in PBS (pH 7.4) solution.

Nanostructured PAMAM dendrimers with the functional group of 256 end amines were chosen as the good scaffolds to assemble dual antibodies, owing to their high payload and multivalent binding effect [20,37]. AntiEpCAM and/or antiSlex antibodies were sequentially conjugated onto the completely carboxylated G6 PAMAM (CC G6) dendrimer surface as previously reported [20]. Fluorescence-labeled dual antibody conjugate was similarly synthesized by using phycoerythrin (PE) linked antiEpCAM (antiEpCAM-PE) and fluorescein isothiocyanate (FITC) linked antiSlex (antiSlex-FITC, i.e., antiSlex and IgG/IgM-FITC antibodies were used together and abbreviated as antiSlex-FITC hereafter), instead (Figure 1a). The resultant antibody conjugates were used for the following cancer metastasis prevention assays including cell binding, cell activity regulation and cell adhesion (Figure 1a). For PE-5A-G6-5S-FITC conjugate in aqueous solution, fluorescence images taken at λ_ex_ 488 and 543 nm demonstrated each antibody was coated on the modified dendrimer surface (Figure 1b). For G6-5A-5S conjugate, field emission scanning electron microscope (FSEM) measurement showed its characteristic morphology of round pie with the particle size of 100 nm (Figure 1c). UV spectra analysis at λ_220 nm_ indicated the successful coating of dendrimers with antibodies in comparison with the non-absorption of CC G6 dendrimers (Figure 1d).

### CTCs bound and captured by the conjugate

Considering colon cancer cell lines including SW480, SW620 and LoVo express different levels of biomarkers (e.g., EpCAM), which was confirmed by the flow cytometry (Additional file 1: Figure S1), the binding and capture capability of antiEpCAM and antiSlex sequentially-conjugated dendrimer conjugate (PE-5A-G6-5S-FITC) to the above EpCAM and Slex-expressing colon cancer cell lines was individually investigated by us.

#### Specificity in recognizing and binding the adherent cells

**Figure 2:**
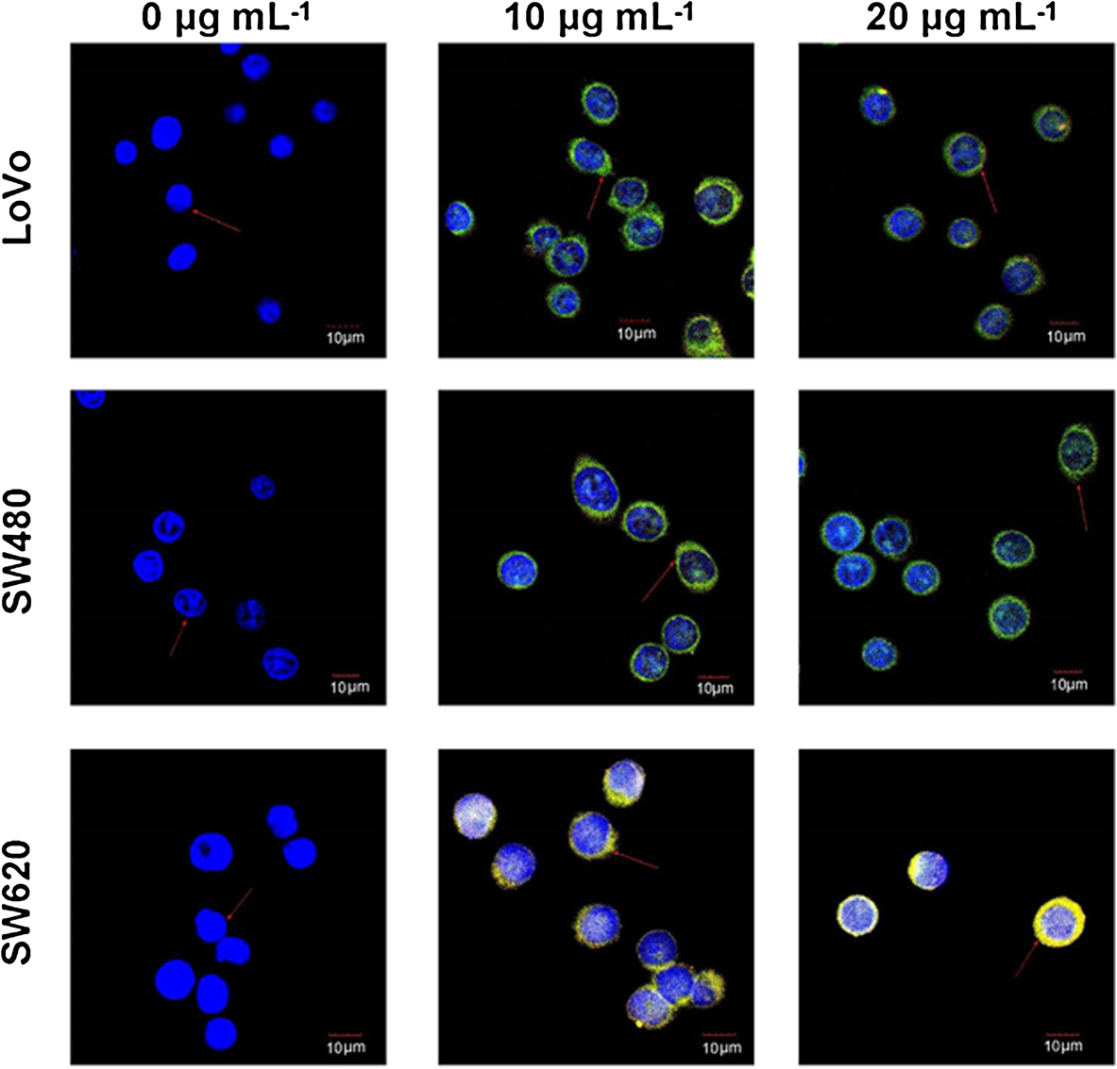
**Fluorescence micrographs of the adherent colon cancer cell lines (SW620, SW480 and LoVo) respectively bound by PE-5A-G6-5S-FITC conjugate at various concentrations (0, 10, 20 μg mL** ^**−1**^ **).** Cell nucleus was stained with blue color (positive to DAPI) while cell membrane was labeled with the merged yellow-green color (positive to both FITC and PE).

Preliminary experiments indicated that G6-5A-5S conjugate could efficiently bind the target cells within 1 h (Additional file 1: Figure S2). To qualitatively evaluate the binding effects of G6-5A-5S conjugate at various concentrations on the three adherent colon cancer cell lines, laser confocal microscope analysis was performed. The cell nucleus was labeled with blue color to distinguish other cell components. Once cells were recognized and bound by PE-5A-G6-5S-FITC conjugate, the merged yellow-green color was displayed in cellular membrane. Fluorescence intensity was concentration-dependently increased with the conjugate increased from 10 to 20 μg mL^−1^. Moreover, fluorescence intensity from the cytomembrane of SW620 cells was more stronger than that from LoVo and SW480 cell lines (Figure 2). It seemed that the conjugate was more internalized in SW620 cell than in SW480 and LoVo cell lines through the double specific antigen-antibody interactions.

#### Efficiency in capturing the suspensory cells

**Figure 3:**
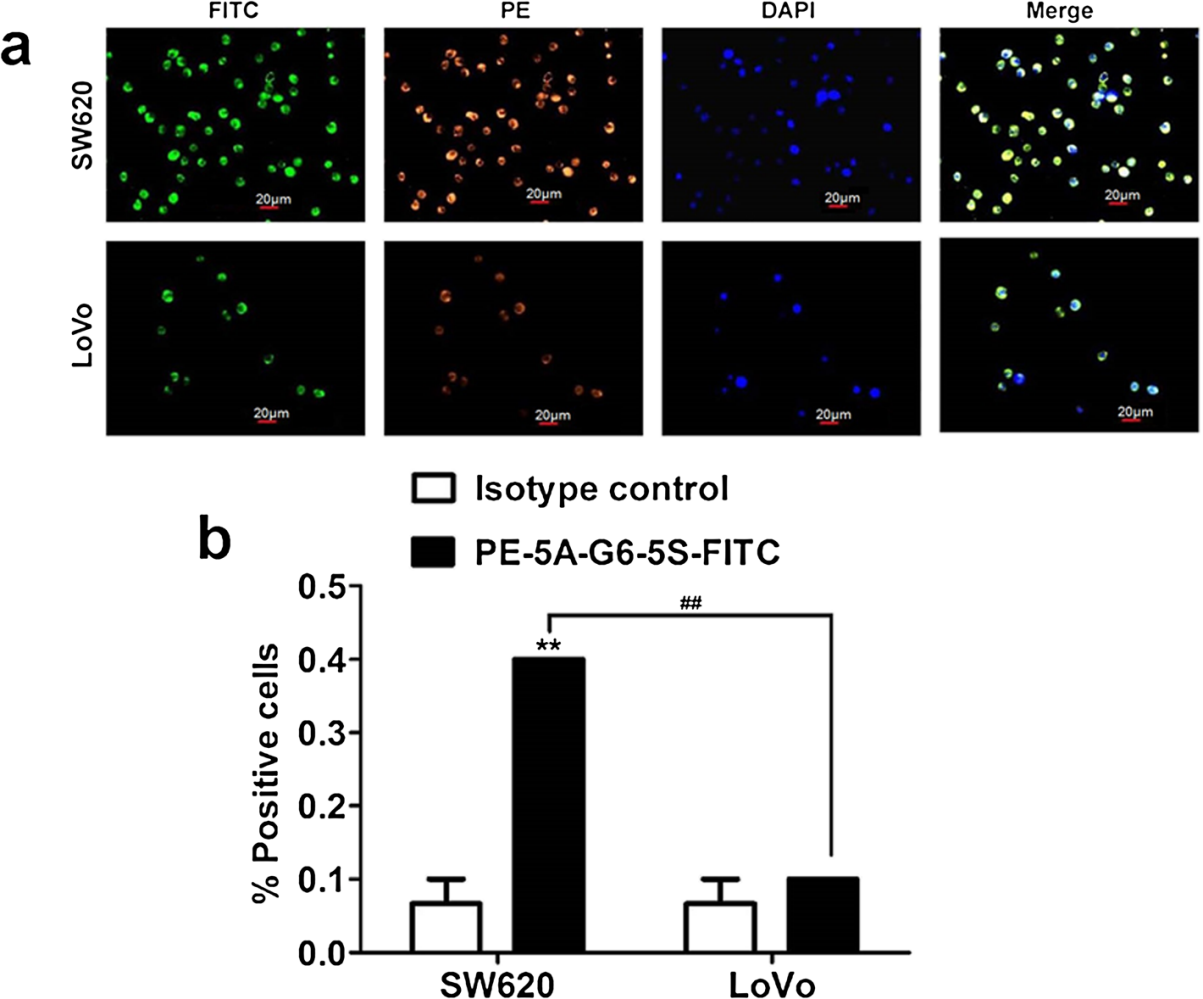
**Qualitative and quantitative analyses of the suspensory SW620 and LoVo cell lines respectively captured by PE-5A-G6-5S-FITC conjugate at the same concentration of 20 μg mL** ^**−1**^ **. a**, Representative fluorescence images of the captured cell lines at different excitation channels. **b**, Flow cytometric analysis of the capture efficiency of the conjugate in comparison with the isotype control.

The metastatic ability of non-adherent CTCs [38] may differ from that of adherent CTCs [39]. To further evaluate the capture capability of PE-5A-G6-5S-FITC conjugate (20 μg mL^−1^) to the suspensory cell lines, fluorescence inverted microscope and flow cytometric analyses were both carried out. The cell lines have identical exposure time (1 h) and baseline of fluorescence intensity. Without the non-specific binding, the conjugate showed the specific receptor-mediated binding to both SW620 and LoVo cell lines, which was seen from the distinct yellow-green fluorescence on cytomembrane. The increased fluorescence intensity was displayed on SW620 cell than on LoVo cell. The number of captured SW620 cells seemed to be more than that of captured LoVo cells in any random visual field (Figure 3a). The capture efficiency of the conjugate was also quantitatively evaluated by the % FITC and PE-positive cells within Q2 quadrant analyzed by the flow cytometry. Relative to the isotype control, the capture efficiency for SW620 cells was 4-fold higher than that for LoVo cells based on the captured numbers (Figure 3b). The enhanced capture efficiency for SW620 cells might be up to the relatively higher expression levels of EpCAM and Slex (Additional file 1: Figure S1).

### Down-regulation of the activity of captured cells by the conjugate

Once cancer cells were captured, the interactions between single or dual antibody conjugates and cell lines were investigated by us in parallel as follows.

#### Inhibition of the proliferation of captured cells

**Figure 4:**
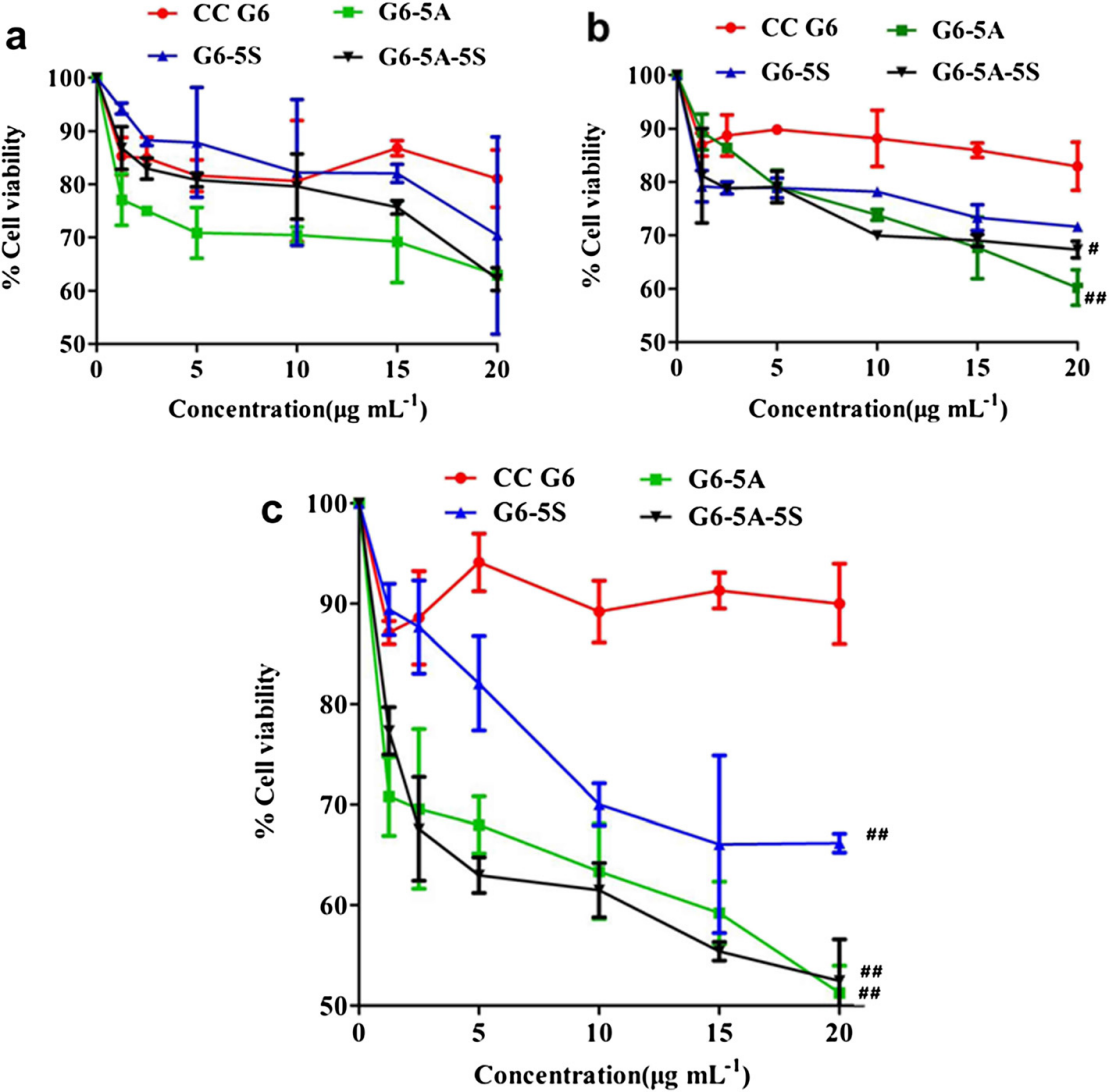
**Decrease in viability of colon cancer cell lines induced by single and dual antibody conjugates at concentrations ranging from 1.25 to 20 μg mL** ^**−1**^ **. a**, SW480 cells; **b**, LoVo cells; **c**, SW620 cells.

The effects of single and dual antibody conjugates (G6-5A, G6-5S, G6-5A-5S) on the proliferation of captured cells were studied first of all. 48 h of binding treatment resulted in the decreased cell viability relative to control. With the concentration of conjugate increased from 1.25 to 20 μg mL^−1^, the cell activity of each cell line was concentration-dependently restrained (Figure 4a-c). The cell viability caused by single and dual antibody conjugates was compared to that by CC G6 dendrimers. CC G6 dendrimers remained more than 80% of cell viability even at the concentration of 20 μg mL^−1^. After single or dual antibody was conjugated onto dendrimer surface, the viability of each cancer cell line was obviously decreased. The single or dual antibody conjugate showed the stronger inhibitory effect on SW620 than on LoVo and SW480 cell lines. For example, the cell activity of SW620, LoVo and SW480 was reduced to 51.24%, 60.22% and 62.93%, respectively, by G6-5A conjugate (20 μg mL^−1^) (Figure 4a-c). It seemed that conjugates produced the inhibitory but not the lethal effects. The high levels of EpCAM and Slex might contribute to the selectivity of the conjugates for SW620 cells. G6-5A-5S and G6-5A conjugates had the stronger capability of restraining the activity of the same cells compared to G6-5S conjugate (Figure 4a-c). The decreased cell activity might be mainly attributed to the presence of EpCAM on cancer cells. The down-regulation of the captured colon cancer cell lines indicated that dual antibody-coated dendrimers may fit into a new class of therapeutic for preventing cancer metastasis by selectively restraining target CTCs rather than non-selectively killing normal and cancer cells.

#### Flow cytometric analysis of cell cycle distribution

**Figure 5:**
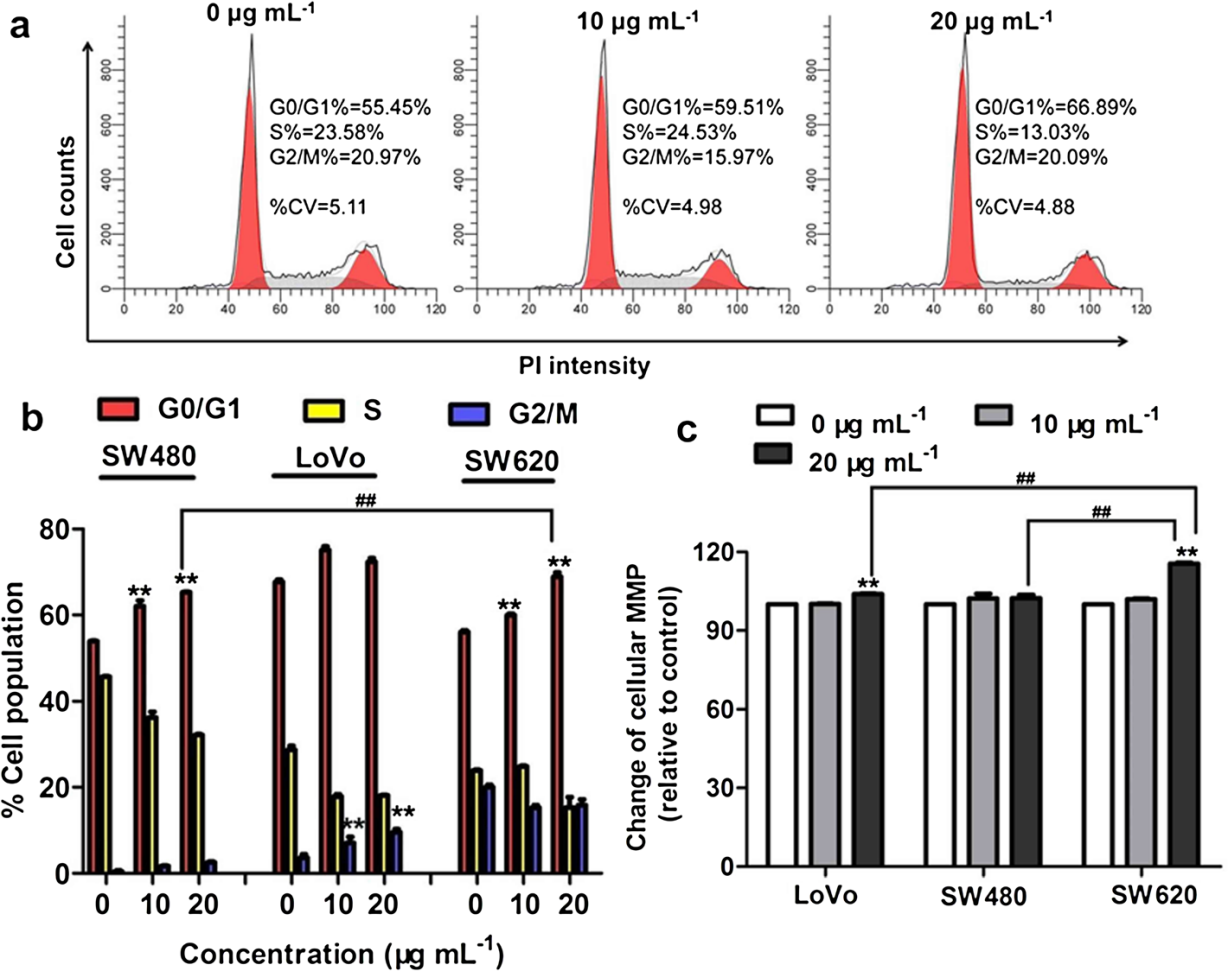
**Cell cycle distribution and cellular MMP of colon cancer cell lines after exposure to dual antibody conjugate G6-5A-5S at 10 and 20 μg mL** ^**−1**^ **. a**, DNA flow cytometric images of the treated SW620 cells with the conjugate. **b**, Percentage of cell population in every stage (G0/G1, S and G2/M). **c**, The influence of conjugate on the cellular MMP. The increased fluorescence intensity of DiOC6(3) usually indicated the decreased MMP.

To further explore how the G6-5A-5S conjugate affected the cell activity, cell cycle distribution was analyzed by flow cytometry. Cell lines after individual incubation with the conjugate for 48 h were stained with propidium iodide (PI) staining to determine the cell population in every phases of G0/G1, S and G2/M. Flow cytometric images showed that the conjugate could cause a concentration-dependent increase in cell population of the G0/G1 phase and a decrease in cell population of the S phase without a significant increase in cell population of the G2/M phase for SW620 cells (Figure 5a). Similar cell cycle distribution was found for SW480 cells. The significance between SW480 and SW620 cell lines indicated the increased inhibitory effect of conjugate on SW620 cells. However, for LoVo cells, the cell population in G2/M phase was concentration-dependently increased and that in S phase was decreased without a significant change in G0/G1 phase (Figure 5b), suggesting that dual antibody conjugate G6-5A-5S mainly arrested SW480 and SW620 cell lines at the G0/G1 stage and LoVo cells at the G2/M stage. The difference in cell cycle distribution might be attributed to the different interaction mechanism between dual antibody conjugate and each colon cancer cell line.

#### Cellular mitochondrial membrane potential (MMP) evaluation

MMP (Δψm) depolarization is a prelude of cell apoptosis. The effects on cellular Δψm induced by various concentrations of G6-5A-5S conjugate (0, 10, 20 μg mL^−1^) were measured with DiOC6(3) (3,3’-Dihexyloxacarbocyanine iodide) staining. The increased fluorescence intensity predicts the decreased Δψm. Flow cytometric analysis showed that fluorescence intensity from the treated SW620 cells was stronger than that from the untreated ones. The cellular Δψm was decreased in a moderate concentration-dependent manner. However, the conjugate at the concentration up to 20 μg mL^−1^ only produced 15% loss of Δψm. In contrast, the MMP of treated SW480 and LoVo cell lines wasn’t significantly affected by the conjugate, indicating the mitochondrial function and electron transport chain activity are kept intact (Figure 5c). It seems like that the dual antibody-coated dendrimer conjugate could result in the change of cellular MMP.

### Inhibition of the adhesion of cancer cells by the conjugate

We performed the related adhesion assays as follows by using the conjugate at the safe concentrations ranging from 1.25, 2.5, 5 to 10 μg mL^−1^ to determine whether the single and dual antibody conjugates could intervene the adhesion of cancer cells to endothelial cells for cancer metastasis prevention.

#### Substrate adhesion analysis

**Figure 6:**
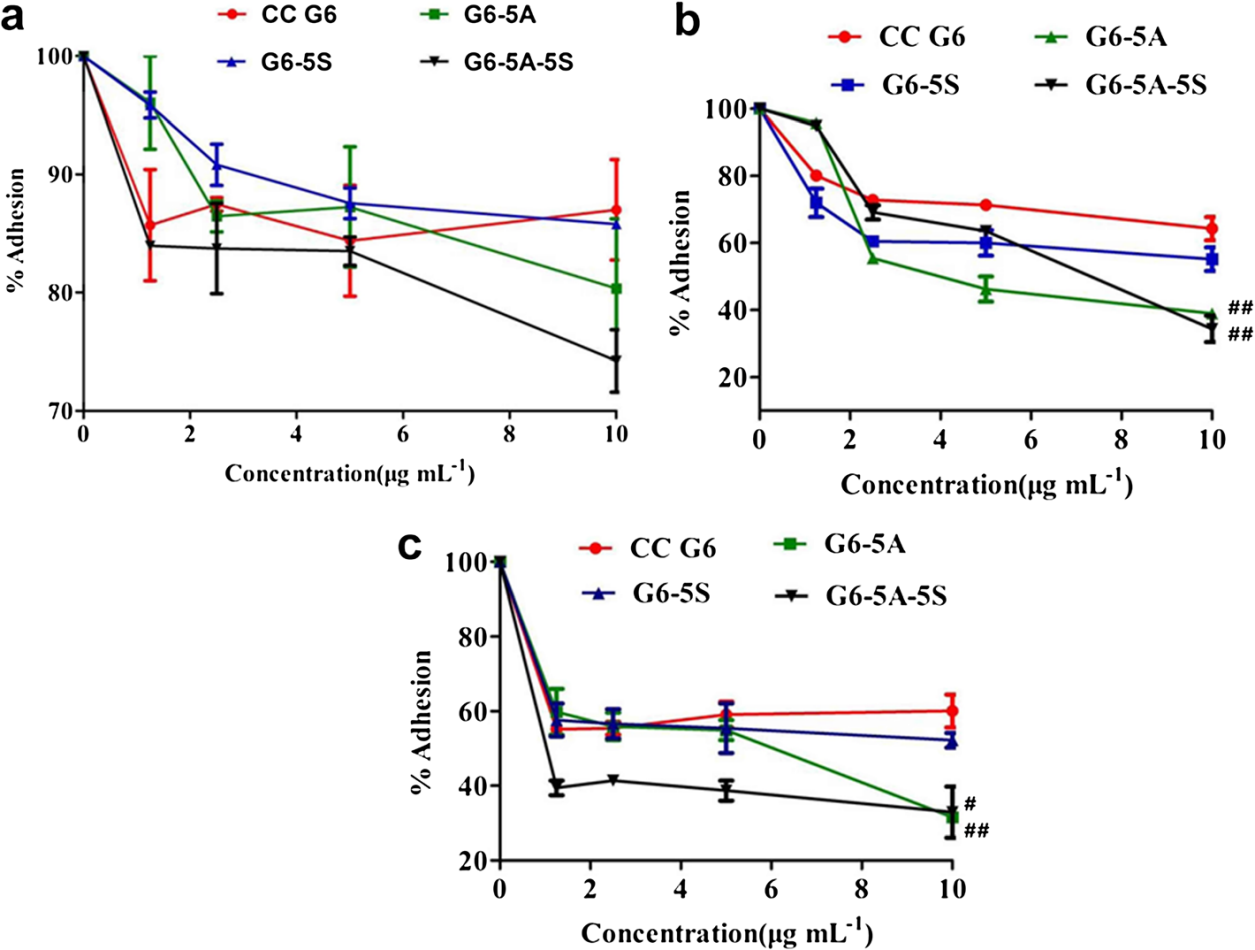
**The concentration-dependent inhibition of single and dual antibody conjugates (from 1.25 to 10 μg mL** ^**−1**^ **) on the adhesion of colon cancer cell lines to Fn-coated substrate. a**, SW480 cells; **b**, LoVo cells; **c**, SW620 cells.

EpCAM and Slex are two adhesion molecules expressed by CTCs not by hematologic cells [40-42]. E-selectin was mainly expressed on the activated endothelial cell surface of blood vessels. The interaction between Slex and E-selectin mediated the hetero-adhesion of CTCs to vascular endothelial cells and the continued survival and proliferation of CTCs [35,36]. Conjugation of antiEpCAM and/or antiSlex antibodies onto the dendrimer surface might effectively interfere the hetero-adhesion of cancer cells to basement membrane and endothelial cells. Similar MTT {[3-(4,5-dimethylthiazol-2-yl)-2,5-diphenyltetrasodium bromide] tetrazolium salt} assay was used to evaluate the inhibitory effects of G6-5A, G6-5S and G6-5A-5S conjugates on the adhesion of colon cancer cell lines to fibronectin (Fn)-coated artificial substrate membrane. CC G6 dendrimers used as the controls were also tested to demonstrate their low effects in adhesion process. In contrast, the antibody conjugates could lead to the reduced adhesion between cancer cells and Fn with concentrations increased from 1.25 to 10 μg mL^−1^. The adhesive percentage of SW480, LoVo and SW620 cell lines was 74.20%, 34.30% and 32.93%, respectively, with the G6-5A-5S conjugate at 10 μg mL^−1^ (Figure 6a-c). It was seen that the conjugate was not effective in blocking the adhesion of SW480 cells but the adhesion of SW620 and LoVo cell lines to Fn. In comparison with single antibody conjugates G6-5A and G6-5S, dual antibody conjugate G6-5A-5S showed the stronger interference ability (Figure 6a-c). For LoVo cells, the mean anti-adhesion efficacy of G6-5S, G6-5A and G6-5A-5S were 44.90%, 61.09% and 65.70%, respectively (Figure 6b), suggesting that antiEpCAM and antiSlex antibodies might play the synergistic effects in inhibiting the adhesion of cancer cells to Fn-coated substrates, and the antiEpCAM was the key factor with this respect.

#### HUVECs adhesion analysis

**Figure 7:**
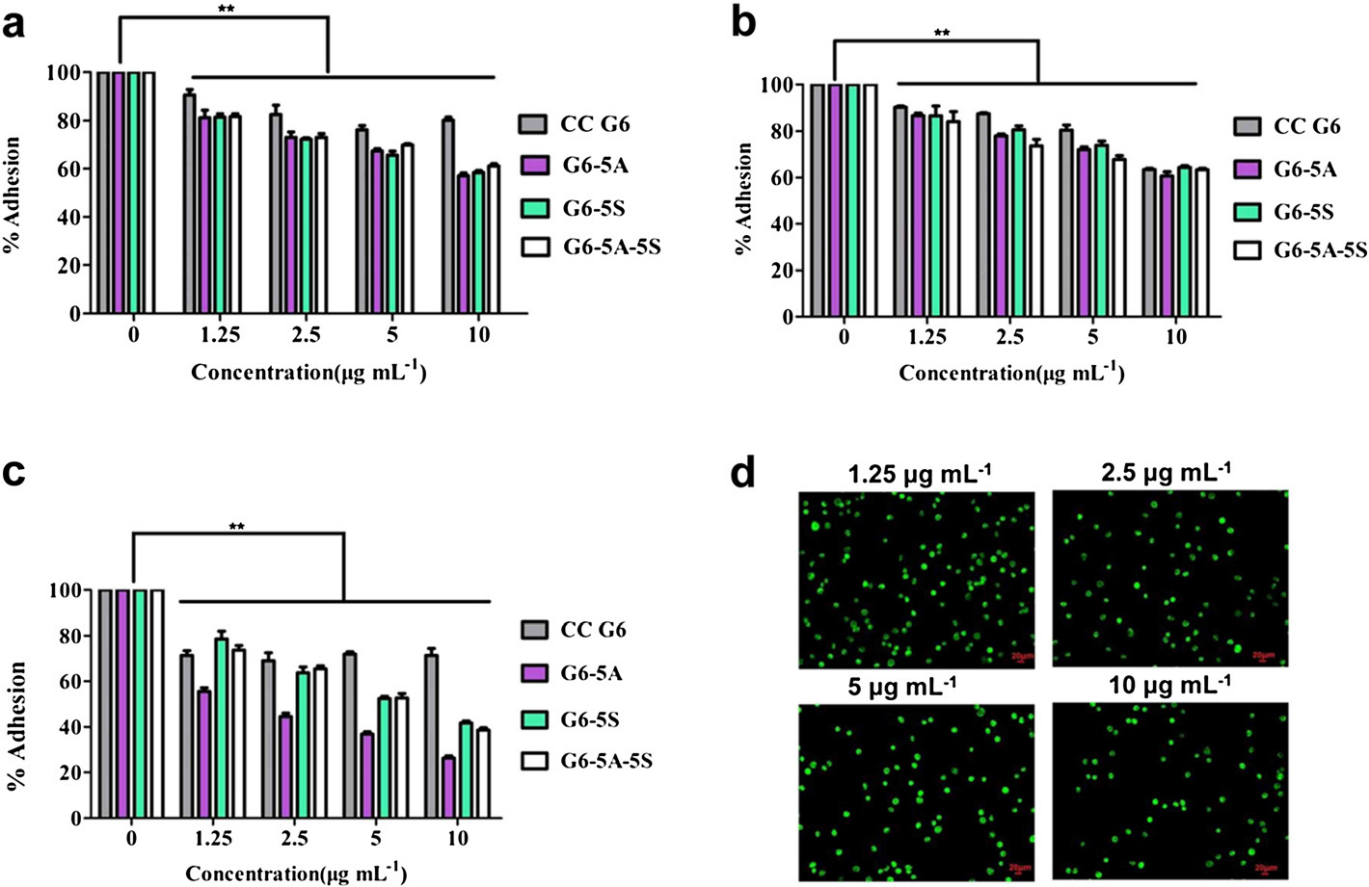
**Inhibition by single and dual antibody conjugates on the hetero-adhesion between colon cancer cell lines and HUVECs concentration-dependently (1.25 to 10 μg mL** ^**−1**^ **). a-c**, The conjugates showed the different capability in interfering with the adhesion of cancer cell lines to HUVECs. **a**, LoVo cells; **b**, SW480 cells; **c**, SW620 cells. **d**, Representative fluorescence images of Rhodamine 123-labeled SW480 cells that adhered to HUVECs when they were treated with different concentrations of G6-5A-5S conjugate.

Hetero-adhesion of CTCs to local vascular endothelium initiates the irreversible cancer metastasis. Using the targeting antibodies-coated nanomaterials to interfere the adhesion process will be a new attempt. In this assay, fluorescence microscopic analysis was performed to assess the anti-adhesion effects of single and dual antibody conjugates (G6-5A, G6-5S and G6-5A-5S). The hetero-adhesion of three colon cancer cell lines to HUVECs was individually inhibited by the conjugates in a concentration-dependent manner (Figure 7a-c), for example, the number of SW480 cells with green fluorescence that adhered to HUVECs was concentration-dependently decreased by G6-5A-5S from 1.25 to 10 μg mL^−1^ (Figure 7d). However, the anti-adhesion effects of the same conjugate varied with different colon cancer cell lines and the capability of single and dual antibody conjugates was also different. It seemed that conjugates displayed the stronger capability to interfere the adhesion between SW620 cells and HUVECs in comparison with the adhesion between other two cell lines and HUVECs. The maximum adhesion percentage of SW620, LoVo, and SW480 by the G6-5A-5S conjugate (10 μg mL^−1^) was 38.60%, 61.11%, and 63.25%, respectively (Figure 7a-c). G6-5A and G6-5A-5S conjugates were superior to G6-5S conjugate in interfering the adhesion of colon cancer cells to HUVECs. The adhesion of SW620 cells to HUVECs was reduced by 73.68% (G6-5A), by 58.19% (G6-5S), and by 61.40% (G6-5A-5S), respectively, at the same concentration of 10 μg mL^−1^ (Figure 7c). The different anti-adhesion capability of the conjugates to each cancer cell line might contribute to the selection of the appropriate conjugate as the specific target for preventing cancer metastasis.

## Discussion

AntiEpCAM and antiSlex collectively-coated dendrimer conjugates were synthesized, for the first time, by employing the surface coating technology [20,43]. The successful surface functionalization was demonstrated both by UV spectra and fluorescence images. FSEM measurement also showed the morphology and size of the antibody-conjugated dendrimers (Figure 1). The average size of G6 PAMAM dendrimer is about 7 nm. After conjugation with antibody, the size of the PAMAM dendrimer quickly grew to 100 nm. The increment in size may be attributed to the increased numbers of the dual antibodies conjugated. Moreover, two different antibodies with the different size, specie, character and charge may also result in the high variance in coating technique and particle size.

Compared to the reported single antibody-coated dendrimers [44,45], dual ones played the synergistic effects in biological functions [46]. After excluding the non-specific binding and cell autofluorescence with the isotype controls, whatever to the adherent or suspensory cells, PE-5A-G6-5S-FITC conjugate displayed the specific recognition and binding affinity (Figures 2 and 3), which might be attributed to the antiEpCAM/EpCAM and antiSlex/Slex double interactions. The conjugate could be internalized into cytomembrane and cytoplasm not organelles with the concentration increased or incubation time prolonged (Figure 2, Additional file 1: Figure S2), which might be caused by the cell endocytosis and the enhanced permeability and retention (EPR) effects of nanomaterials. The interactions between chemotherapeutics and cancer cells were studied by us [10,29,47], so we have a good understanding of the molecular characterization of cancer cells. Though dendrimers with carboxyl ending groups were reported to be less toxic and more compatible than dendrimers with amino terminal groups [48,49], whether dendrimers coated with antiEpCAM and/or antiSlex affected the activity of captured cells was investigated. Our studies indicated that G6-5A and G6-5A-5S conjugates decreased the viability of colon cancer cell lines (especially SW620 cells) more significantly than G6-5S conjugate (Figure 4). We further explored the regulation mechanism by which G6-5A-5S conjugate blocked the cell cycle and reduced the cellular MMP in a modest concentration-dependent way (Figure 5). The result about cell activity regulation was in agreement with what we have previously reported [46,50]. Taken into account that the interaction between Slex and E-selectin mediated the adhesion of CTCs to endothelial cells, the intervention effects of the conjugates were further explored. Adhesion assays showed that both single and dual antibody conjugates effectively inhibited the hetero-adhesion between each colon cancer cell line and HUVECs/Fn-coated substrates (Figures 6 and 7). G6-5A and G6-5A-5S conjugates had the better anti-adhesion effects than G6-5S conjugate. In comparison with SW480 and LoVo cell lines, SW620 cells were more significantly affected by the conjugates, indicating that antiEpCAM/EpCAM interaction played the critical roles in the binding, regulation and adhesion processes (Additional file 1: Figure S1). Superior to the detection function, the dual biological functions of dual antibody-coated nanomaterials, including anti-proliferation and anti-adhesion effects, might interfere the critical points of initiating cancer metastasis.

## Conclusions

In conclusion, the present study firstly synthesized the dual antibody-coated dendrimers with or without fluorescence labeling, and fully characterized their physicochemical properties. The dual antibody conjugates bound or captured the colon cancer cell lines with the enhanced affinity and specificity, and exhibited the superiority to their single counterparts in the restraint of cell activity and in the inhibition of the hetero-adhesion of cancer cells to endothelial cells. These newly-found biological functions of the re-engineered nanomaterials with antibodies may aid in designing new strategy to effectively prevent cancer metastasis by targeting the biomarkers-abundant cancer cells.

## Materials and methods

### Synthesis and characterization of antiEpCAM- and antiSlex- coated dendrimer conjugates with or without fluorescence labeling

To investigate the dual roles of dendrimer-antibody conjugates in cancer metastasis prevention, PAMAM dendrimers with the ethylenediamine core [generation 6 (G6), theoretical MW 624,00 Da] provided by Shandong Weihai Chenyuan New Silicone Materials, Co. Ltd were firstly surface-modified and sequentially conjugated with two fluorescence or non fluorescence-labeled antibodies. Briefly, G6 PAMAM dendrimers with amine ends of 256 (100 mg, 1.60 μmol) were dissolved in 2 mL DMSO, and respectively reacted with 410 mg succinic anhydride (SA) (4.1 mmol, ten molar excess) under vigorous stirring overnight. The obtained CC G6 dendrimers were dialyzed against DDI water to remove the unreacted molecules as well as organic solvents before lyophilization.

The dual antibody-conjugated dendrimer conjugate was synthesized by employing the 1-ethyl-3-(3-dimethylaminopropyl) carbodiimide (EDC) catalytic method and designated as G6-5A-5S based on the reaction molar ratio of 1 dendrimers to 5 antiEpCAM to 5 antiSlex. AntiSlex (MW150KDa), IgG/IgM-FITC, antiEpCAM-PE were provided by BD company, and antiEpCAM (MW150 KDa) was purchased from Sigma-Aldrich and Abcam (Hong Kong) Ltd. CC G6 dendrimers (0.55 μg, 7.9 pmol) dissolved in 2 mL phosphate-buffered saline (PBS) were activated using EDC (75.8 ng, 395.3 pmol, 50 molar excess) and N-hydroxysuccinimide (NHS) (45.5 ng, 395.3 pmol, 50 molar excess) at room temperature for 1 h. The activated dendrimers were reacted with the combined antiEpCAM (39.5 pmol, 5 molar excess) and antiSlex (39.5 pmol, 5 molar excess) under vigorous stirring overnight. The single antibody-coated dendrimer conjugates were similarly synthesized and denoted as G6-5A and G6-5S. There were approximately two aEpCAM molecules in one G6-5A conjugate and six aSlex in one G6-5S conjugate according to the UV analysis. The fluorescence-labeled dual antibody conjugate PE-5A-G6-5S-FITC was also synthesized and designated following the similar procedure just by using antiEpCAM-PE and antiSlex-FITC (antiSlex and IgG/IgM-FITC were used together) antibodies, instead. The activated CC G6 dendrimers were reacted with antiSlex-FITC for 12 h, then with antiEpCAM-PE for another 12 h. All the reactions were conducted under vigorous stirring overnight in dark. Finally, all the conjugates were purified via dialysis (10,000 MWCO) against DDI water overnight before lyophilization. Dendrimers with large surface functional groups were almost able to assemble with all of the antibodies as one entity at the above designed molar ratios. Transmembrane dialysis was used to remove the intermediates and small molecules.

The presence of antibody or fluorescence-labeled antibody onto the dendrimer surface was respectively confirmed by the UV absorption value at λ_220 nm_ (Quawell 5000 UV–vis Spectrophotometer, America) and the merged fluorescence intensity at FITC λ_ex_ 488 nm, λ_em_ 500–535 nm and PE λ_ex_ 568 nm, λ_em_ 560–660 nm (Olympus FluoView 1000). The morphological property and particle size of G6-5A-5S conjugate were determined by FSEM measurement.

### Cell culture

Human colorectal carcinoma cell lines including SW480, SW620 and LoVo were purchased from the Type Culture Collection of the Chinese Academy of Sciences, Shanghai, China and kept in a minimal number of passages, then cultured in RPMI 1640 medium supplemented with 10% heat-inactivated fetal calf serum (FCS) and 1% penicillin/ streptomycin (P/S). HUVECs were obtained from the fresh human umbilical cords of new-born babies with 1 mg mL^−1^ of collagenase in PBS and cultured by us with M199 medium supplemented with 20% fetal bovine serum (FBS), 100 μg mL^−1^ endothelial cell growth supplement (ECGS), 50 μg mL^−1^ heparin sodium and 1% P/S in a culture flask coated with 0.2% gelatin after some necessary pretreatment [10,51]. All the cell lines above were grown in a humidified atmosphere of 5% CO_2_ at 37°C for the subsequent experiments. HUVECs were used for no more than six passages.

### Flow cytometric procedures

A Becton Dickinson (BD) multiparametric fluorescence-activated cell sorting (FACS) Aria III with laser excitation set at 488 was used for flow cytometric analysis. According to the forward versus side scatter histograms, gating strategy was used to set P1 gate for determining the target colon cancer cell lines. Fluorescence signals derived from PI (or PE) and DiOC6(3) (or FITC) were respectively detected through 585 and 530 nm bandpass filters. Side angle scattered light (SSC) versus PI histogram displayed the cell cycle distribution, SSC versus PE (or FITC) histogram showed the expression levels of biomarkers, SSC versus DiOC6(3) histogram revealed the cellular MMP, and PE versus FITC dot plots showed the captured cell numbers by the synthesized conjugate. All the data were acquired based on the collected 10,000 cells satisfying the light scatter criteria and analyzed using the BD FACS Diva software provided with the system.

### CTCs binding and capture assays

To explore the binding and capture capability of fluorescence-labeled dual antibody conjugate PE-5A-G6-5S-FITC at various concentrations (0, 10, 20 μg mL^−1^) to the adherent and suspensory colon cancer cell lines, the optimal incubation time was in advance determined according to the analysis of time-response cell capture assay shown in Additional file 1. The preliminary result showed that 1 h-binding time was *sine qua non* for the quick and efficient cell capture.

#### Binding to the adherent cells

Cell lines at the density of 10^5^/mL were cultivated on 35 mm dishes with glass coverslips in the bottom, and individually treated with PBS containing 1% bovine serum albumin (BSA) (1% PBSA) for 30 min. After 1 h of co-incubation with PE-5A-G6-5S-FITC conjugate at various concentrations (0, 10, 20 μg mL^−1^) in a humidified atmosphere of 5% CO_2_ at 37°C, cell lines were washed with PBS to remove the unbound conjugate, and fixed with stationary liquid (V_methanol_:V_acetone_ = 7:3) for 1 min, then stained with 10 μg mL^−1^ of nuclei stain dihydrochloride (DAPI) solution for 15 min. Finally, cell lines were covered with serum-free medium for images taken by an Olympus FluoView 1000 laser confocal microscope respectively in the channel of DAPI, Alex Fluor 488 and 568.

#### Capturing the suspensory cells

To evaluate the efficiency of PE-5A-G6-5S-FITC conjugate at capturing the colon cancer cell lines, SW620 and LoVo cell lines at the density of 10^6^/mL were suspended in each tube. Cell lines were treated with 1% PBSA, then with 20 μg mL^−1^ of PE-5A-G6-5S-FITC conjugate for 1 h at 37°C water bath. Cell lines without the treatment of conjugate were incubated with immunoglobulins labeled with PE or FITC in the similar way as isotype controls. After washing and centrifugation, the unbound conjugates or antibodies were abandoned. Cell lines suspended with PBS buffer were directly analyzed on a BD FACS Aria III analyzer with laser excitation set at 488 nm or further stained with Hoechst 33258 (labeling the nucleus) for analysis with a fluorescence inverted microscope (Axio Observer A1, Zeiss, Germany).

### Restraining the captured CTCs for preventing cancer metastasis

#### Cell viability

To investigate how the single and dual antibody conjugates (G6-5A, G6-5S and G6-5A-5S) affected the cell proliferation, MTT analysis was conducted as we previously described. The effect of completely-carboxylated G6 dendrimers on cell activity was also tested. Cell lines at the density of 5 × 10^3^-1 × 10^4^ cells/mL were cultivated on the 96-well plates with 1640 medium. When grew in the confluence of 70%-80%, cell lines were individually exposed to the conjugates at various concentrations (0, 1.25, 2.5, 5, 10, 15, 20 μg mL^−1^) for 48 h. Then, 100 μL of serum-free medium containing 1 mg mL^−1^ MTT solution was added to incubate for another 4 h. Finally, the supernatant was aspirated and 150 μL of DMSO was added to each well to dissolve the water-insoluble blue formazan. The viability of each cell line induced by the conjugates was determined based on the optical absorption value at the wavelength of 570 nm (A_570 nm_) and expressed as A_570 nm_ of the treated group divided by that of the control group.

#### Cell cycle distribution

To further discuss the effects of the antibody conjugates (e.g., G6-5A-5S) on the cell population distribution in every phases (G0/G1, S, and G2/M), PI staining experiment was performed at 37°C as the kit instructions. Cell lines were cultivated in 6-well plates overnight, and incubated with various concentrations of G6-5A-5S conjugate (0, 10, 20 μg mL^−1^) for 48 h. Then cell lines were trypsinised and washed with ice-cold PBS for three times. After fixed with 70% ice-cold ethanol overnight at −20°C, cell lines were washed and stained with PI solution at 37°C for 15 min. Finally, data acquisition and analysis were performed on a BD FACS Aria III flow cytometer and DNA integration software mflt32, respectively.

#### Cellular MMP

Depolarization of cellular MMP usually predicts the starting of cell apoptosis. In this assay, DiOC6(3) (a lipotropy cationic fluorescent dye) staining was used to determine the change of MMP in colon cancer cell lines. Increment of fluorescence intensity with the accumulation of DiOC6(3) in mitochondria was accompanied with the descent of MMP. After exposure to the antibody conjugates (e.g., G6-5A-5S) at various concentrations (0, 10, 20 μg mL^−1^) for 48 h, cell lines were trypsinized and collected after centrifugation. 500 μL of DiOC6(3) (2 nM) working solution was individually added into each tube, and kept at 37°C water bath for 20 min. The cellular MMP (Δψm) was finally assessed according to the fluorescence intensity of DiOC6(3) examined by the flow cytometry (BD FACS Aria III).

### Inhibiting the adhesion of captured CTCs to endothelial cells for preventing cancer metastasis

#### Blocking the adhesion of cancer cells to Fn-coated substrates

Adhesion of CTCs to extracellular matrix (ECM) was a critical step in the process of cancer metastasis. Colon cancer cell lines were usually used as CTC models. In cell adhesion assays, CC G6 dendrimers were used as the control. Fn-coated substrates were prepared as the ECM to test the capability of single and dual antibody conjugates (G6-5A, G6-5S, G6-5A-5S) in interfering the adhesion of colon cancer cell lines to ECM. First of all, 10 ng mL^−1^ of Fn was pre-coated on the substrates of 96-well plate overnight, then discarded and sealed up with 2% PBSA for 30 min. 100 μL of the mixtures of cell lines and conjugate at various concentrations (0, 1.25, 2.5, 5, 10 μg mL^−1^) were added onto each well for 1 h of incubation. The post-processing was similar to that described in MTT assay. 100 μL of serum-free medium containing 1 mg mL^−1^ MTT solution was used for another 4 h. After the supernatant was aspirated, 100 μL of DMSO was added to each well to dissolve the water-insoluble blue formazan. The optical density was read on an ELISA reader at a wavelength of 570 nm to determine the abilities of the conjugates to interfere with the adhesion. The relative adhesion (%) was finally evaluated by the A_570 nm_ in the treated group compared to that in the control group.

#### Blocking the adhesion of cancer cells to HUVECs

Adhesion of CTCs to vascular endothelium was another crucial starting point of cancer metastasis. HUVECs instead of vascular endothelial cells were used for adhesion assay in vitro. After grew in the confluence of 100% on the 24-well plates, HUVECs were pre-treated with 1 ng mL^−1^ cytokine IL-1β for 4 h followed by individually incubated with the mixture of rhodamine 123-labeled colon cancer cell lines and single or dual antibody conjugates (G6-5A, G6-5S, G6-5A-5S) at various concentrations (0, 1.25, 2.5, 5, 10 μg mL^−1^) for 1 h. After removal of the non-adhered cells with PBS washing, ten visual fields were randomly selected and taken images by a fluorescence inverted microscope (Axio Observer A1, Zeiss, Germany). The capability of the conjugates in inhibiting the adherence of cancer cell lines to HUVECs was determined by counting the numbers of fluorescence-labeled cells that adhered to HUVECs in sample groups relative to those in the control group.

### Statistical analysis

Every experiment was performed independently and repeated at least three times. Data were expressed as the means ± standard deviations (SD). Statistical analysis was done by Student’s t-test and one-way analysis of variance (One-ANOVA). Multiple comparisons of the means were made through One-ANOVA analysis and demonstrated by the least significance difference (LSD) test (IBM SPSS Statistics 19.0). The symbol of * and ** represented the comparison between sample and control, while ^**#**^ and ^**##**^ represented the comparison between any two samples. A probability value of <0.05 was considered significant (* and ^**#**^), and <0.01 was considered extremely significant (** and ^**##**^).

## Additional file

Additional file 1:
**Biomarkers expression and Time-response cell capture.**

## Abbreviations

CTCs: Circulating tumor cells
Fn: Fibronectin
HUVECs: Human umbilical vein endothelial cells
CAP-NO: S-nitrosocaptopril
EpCAM: Epithelial cell adhesion molecule
AntiEpCAM: EpCAM antibody
PE: Phycoerythrin
AntiEpCAM-PE: PE linked antiEpCAM
Sialyl Lewis X (Slex): Saliva acidifying louis oligosaccharides
AntiSlex: Slex antibody
FITC: Fluorescein isothiocyanate
AntiSlex-FITC: FITC linked antiSlex
PAMAM: Polyamidoamine
CC G6: Completely carboxylated G6 PAMAM
SA: Succinic anhydride
EDC: 1-ethyl-3-(3-dimethylaminopropyl) carbodiimide
NHS: N-hydroxysuccinimide
FSEM: Field emission scanning electron microscope
FCS: Fetal calf serum
P/S: Penicillin/ streptomycin
FBS: Fetal bovine serum
ECGS: Endothelial cell growth supplement
SSC: Side angle scattered light
BD: Becton Dickinson
FACS: Fluorescence-activated cell sorting
PBS: Phosphate-buffered saline
BSA: Bovine serum albumin
1% PBSA: PBS containing 1% BSA
PI: Propidium iodide
DAPI: Dihydrochloride
MTT: [3-(4,5-dimethylthiazol-2-yl)-2,5-diphenyltetrasodium bromide] tetrazolium salt
DiOC6(3): 3,3’-Dihexyloxacarbocyanine iodide
MMP: Mitochondrial membrane potential
ECM: Extracellular matrix
EPR: Enhanced permeability and retention
SD: Standard deviations
One-ANOVA: One-way analysis of variance
LSD: Least significance difference
