# Supplementary material for: Exploring cancer metastasis prevention strategy: interrupting adhesion of cancer cells to vascular endothelia of potential metastatic tissues by antibody-coated nanomaterial
Source: J Nanobiotechnology. 2015 Feb 3;13:9. doi: 10.1186/s12951-015-0072-x (PMC4320453; doi:10.1186/s12951-015-0072-x)
Supplement: Additional file 1: — Biomarkers expression and Time-response cell capture. [file 12951_2015_72_MOESM1_ESM.doc]

**Exploring cancer metastasis prevention strategy: interrupting adhesion of cancer cells to vascular endothelia of potential metastatic tissues by antibody-coated nanomaterial**

Jingjing Xie**^1^**, Haiyan Dong**^1^**, Hongning Chen**^1^**, Rongli Zhao**^1^**, Patrick J. Sinko**^2^**, Weiyu Shen**^1^**, Jichuang Wang**^1^**, Yusheng Lu**^1^**, Xiang Yang**^1^**, Fangwei Xie**^3^**, Lee Jia**^1, 4^ ***

**^1^**Cancer Metastasis Alert and Prevention Center, College of Chemistry, Fuzhou University, Fuzhou 350002, China.

**^2^**Rutgers, The State University of New Jersey, 160 Frelinghuysen Road, Piscataway, NJ, 08854, USA.

**^3^**Department of Medicine Oncology, East Hospital of Xiamen University, Fuzhou 350004, China.

**^4^**Biopharmaceutical photocatalysis, State Key Laboratory of Photocatalysis on Energy and Environment, Fuzhou University, Fuzhou 350002, China.

***Corresponding Author:** Lee Jia ([cmapcjia1234@163.com](mailto:cmapcjia1234@163.com) or [pharmlink@gmail.com](mailto:pharmlink@gmail.com)), 523 Industry Road, Science Building, 3FL., Fuzhou University, Fuzhou, Fujian, China, 350002. Phone and Fax: +86-0591-8357-6912.

**Supplementary information**

**Methods**

**Cell biomarkers expression**

To determine the expression levels of EpCAM on the surfaces of three colon cancer cell lines, cells at the density of 10^6^/ mL in each tube were individually treated with 1% PBSA for 30 min excluding the non-specific binding, and then washed with cold PBS for three times. The collected cells were incubated with PE-labeled monoclonal antibody (mAbs) to EpCAM antigens (antiEpCAM-PE) for 1 h at 37 ^0^C. The unbound antibodies were removed after three times of washing with PBS and centrifugation. Finally, all the cells were collected and resuspended with PBS for flow cytometric analysis. Data were expressed as the mean fluorescence intensity or the percentage of PE -positive cells after Immunoglobulins labeled with PE as isotype controls were used to exclude the autofluorescence and non-specific binding.

**Time-dependent cell binding behaviors**

Cell lines were inoculated on the 96-well plates at the final density of 5×10^3^-1×10^4^ cells/ mL. When grew in the confluence of 70-80%, cell lines treated with 1% PBSA were individually exposed to 20 μg mL^-1^ of PE-5A-G6-5S-FITC conjugate at time intervals. After co-incubation for different times (0, 0.5, 1, 2, 4 h) in the atmosphere of 5% CO_2_ at 37 ^0^C, cell lines were washed excluding the unbound conjugate and covered with serum-free medium for fluorescent measurements taken by a Tecan Infinite M200 pro multifunctional microplate reader.

**Results**

**Expression levels of EpCAM on three colon cancer cell lines**

Biomarkers are usually over-expressed on cancer cell surface not on normal cell surface. The expression levels of EpCAM on colon cancer cell lines including SW620, SW480 and LoVo were respectively investigated by flow cytometry. After normalization of the fluorescence intensity using the corresponding isotype control, cell lines treated with antiEpCAM-PE displayed more than 60% positive rate. Moreover, SW620 had higher expression levels than LoVo and SW480 (Supplementary Fig. 1). The expression levels of EpCAM on the cell surface will largely contribute to the capture specificity and binding stability of targeting antibody-coated dendrimers to each cell line.

**
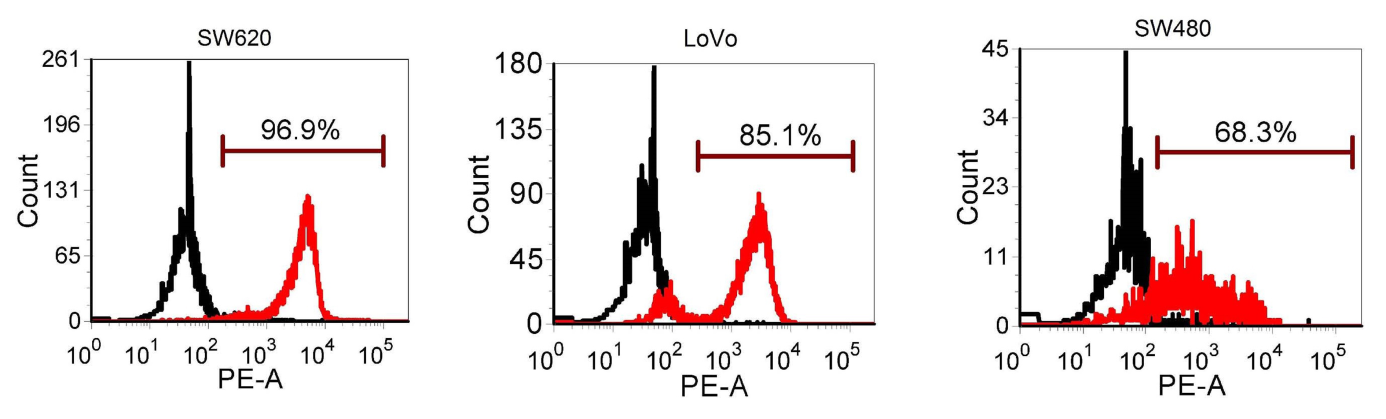
**

**Supplementary Figure 1** Flow histogram of the expression levels of EpCAM biomarker on three colon cancer cell lines (SW620, LoVo and SW480).

**Capture efficiency of the conjugate in response to different time exposures**

The time course of the internalization of PE-5A-G6-5S-FITC conjugate in each cell line was determined both by FITC and PE intensities (Supplementary Fig. 2). After incubation with the conjugate (20 μg mL^-1^) at time intervals (0.5-4 h), fluorescence intensity was respectively read at FITC λ_ex_ 488 nm, λ_em_ 500-535 nm and PE λ_ex_ 543 nm, λ_em_ 560-660 nm. For SW480 and SW620 cell lines, the highest fluorescence intensities of FITC and PE were reached at the time point of 2 h, then gradually decreased until sharply increased at 8 h. Similar phenomenon was found on LoVo cells except that the highest fluorescence intensity was obtained at the time point of 1 h. Taken the cell activity and binding efficiency into account, the 1 h-incubation time was determined.

**
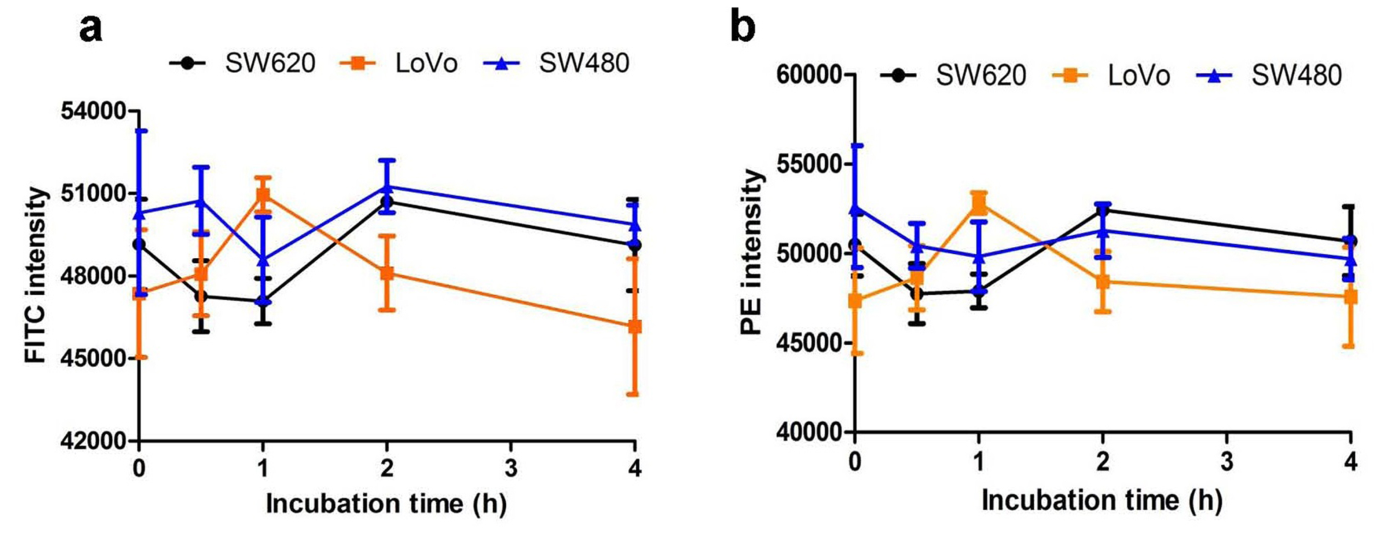
**

**Supplementary Figure 2** Time course-response fluorescence intensities (**a**, FITC; **b**, PE) of the bound colon cancer cell surface by PE-5A-G6-5S-FITC conjugate at the concentration of 20 μg mL^-1^.
